# Fracture of the Lower Jaw Treated Successfully

**Published:** 1852-09

**Authors:** E. McArthur


ART. VI.—*Fracture of the Lower Jaw treated successfully.* By E. McARTHUR, M.D.

MAY 15th, I was called to see Peter Fox, a teamster, who had been run over by a loaded wagon. I found him with three ribs fractured, shoulder badly bruised, right ear and the nose *crushed*, face and one side of the cheek partially *grained*, and a longitudinal fracture of the lower jaw extending from the anterior of the right cuspid to the angle of the jaw on the same side—caused by one or both wheels passing over the mouth. The alveolar arch or process of the bone was separated from the body retaining only a partial connection at the angle. The integuments covering the alveolar process internally and externally, were somewhat bruised and lacerated.

I passed a silver wire between the two middle incisors, and carried it around between the two bicuspid of the fractured part of the jaw, and bringing together the ends twisted them one around the other, until I had got the full strength of the wire, thus holding the fractured portion firmly to the body of the bone.

In four weeks' time the wire was removed, the jaw having become firm and completely ossified.

The treatment for the fractured ribs and bruised shoulder was simply bandaging the body, the application of volatile liniment, cathartics, venesection, expectorants, &c.

I desire more particularly to call attention to the peculiarity of the fractured jaw, its simple treatment, and its complete restoration to a sound and healthy condition.

